# Current Immunotherapies for Sarcoma: Clinical Trials and Rationale

**DOI:** 10.1155/2016/9757219

**Published:** 2016-09-14

**Authors:** Demytra Mitsis, Valerie Francescutti, Joseph Skitzki

**Affiliations:** ^1^Department of Medical Oncology, Roswell Park Cancer Institute, Buffalo, NY 14263, USA; ^2^Department of Surgical Oncology, Roswell Park Cancer Institute, Buffalo, NY 14263, USA; ^3^Department of Immunology, Roswell Park Cancer Institute, Buffalo, NY 14263, USA

## Abstract

Sarcoma tumors are rare and heterogeneous, yet they possess many characteristics that may facilitate immunotherapeutic responses. Both active strategies including vaccines and passive strategies involving cellular adoptive immunotherapy have been applied clinically. Results of these clinical trials indicate a distinct benefit for select patients. The recent breakthrough of immunologic checkpoint inhibition is being rapidly introduced to a variety of tumor types including sarcoma. It is anticipated that these emerging immunotherapies will exhibit clinical efficacy for a variety of sarcomas. The increasing ability to tailor immunologic therapies to sarcoma patients will undoubtedly generate further enthusiasm and clinical research for this treatment modality.

## 1. Introduction 

Some of the earliest evidence for antitumor immune reactivity was noted in sarcoma tumors dating well over a hundred years ago. The pioneering work performed by William B. Coley demonstrated the ability of a patient's own immune system to reject multiple metastatic sarcoma tumors by injecting the tumors with a live preparation of streptococcal organisms designated as “Coley's toxin” [1]. Coley noted objective antitumor responses not only in the injected lesion, but also at noninjected distant tumor sites suggesting an abscopal effect. Due to toxicity, Coley refined his toxin to include heat-inactivated bacteria and over 1000 cases were recorded with half of the patients demonstrating some degree of response [2]. With unpredictable toxicities and somewhat heterogeneous toxin preparations, Coley's toxin was relegated as a historic curiosity. However, the activation of a patient's endogenous antitumor immune response, the ability to generate distant abscopal antitumor effects, and intratumoral injection for local tumor control are the cornerstones for the current resurgence in cancer immunotherapy. While the majority of these newer immunotherapy strategies have involved prototypical “immunogenic tumors” like melanoma, there is a growing body of evidence that all tumors may be susceptible including sarcomas [3]. A rate limiting step in the field of immunotherapy for sarcomas is their intrinsic nature of being relatively rare and heterogeneous. Despite these obstacles, data for immunotherapy of sarcoma is accumulating including the use of vaccines, checkpoint inhibitors, and adoptive immunotherapy. Furthermore, recent advances in these modalities are expected to be translated into future clinical trials of sarcoma immunotherapy.

## 2. Vaccine Therapy

Tumor vaccines stimulate an immune response via specific tumor epitopes while increasing activation of tumor specific T-cells and B-cells [4]. Vaccine therapy has been the most investigated immunotherapy modality in soft tissue sarcomas (STS) and ongoing studies continue to explore its future role in the landscape of STS therapy. Sarcomas are ideal vaccine targets given the heterogeneity of tumor types and their wide expression of immunogenic proteins and antigens, including the cancer-testes antigen family (NY-ESO-1, MAGE-A3, PRAME, and LAGE-1), gangliosides (GM2, GD2, and GD3), sarcoma specific-fusion proteins (SSX, FOXO1, EWSR1, and TLS CHOP), and heat shock proteins [5]. Various vaccine strategies have been used including targeting the aforementioned antigens, tumor lysate, dendritic cells pulsed with antigen, and, most recently, heat shock proteins [6].

The cancer-testes antigen NY-ESO-1 represents an attractive target in STS vaccine establishment. It is expressed in 80% of synovial sarcomas and nearly all myxoid/round cell liposarcomas [7, 8]. Given the prevalence of this antigen, many ongoing STS immunotherapy trials focus on NY-ESO-1 positive tumors. Results are pending from a recently completed phase I/II study that examined the administration of recombinant NY-ESO-1 with immune stimulants (Resiquimod and/or Poly-ICLC) in patients with malignancies known to express NY-ESO-1, including sarcoma (NCT00948961). A similar active study explores the immune response of recombinant NY-ESO-1 antigen and an adjuvant, GLA-SE (NCT02015416).

Another ongoing phase I trial examines the role of recombinant NY-ESO-1 via intranodal injection with or without the mammalian target of rapamycin (mTOR) inhibitor sirolimus in NY-ESO-1 expressing tumors (NCT01522820). Inhibition of mTOR has been shown to strongly influence vaccine induced CD8+ T-cell response, leading to greater antitumor efficacy [9]. The results of a phase II trial from Memorial Sloan-Kettering Cancer Center utilizing a vaccine derived from autologous tumor cell heat shock protein complex (NCT00005628) in patients with recurrent soft tissue sarcoma are also pending.

Gangliosides are richly expressed in sarcomas, making them an exceptional target for vaccine therapy. Among them, GM2, GD2, and GD3 are the most abundant [4, 10]. A randomized double-blinded, multicenter phase II trial from Memorial Sloan-Kettering Cancer Center explored the use of trivalent ganglioside vaccine targeting GM2, GD2, and GD3 combined with immunological adjuvant OPT-821 versus OPT-821 plus placebo in patients with metastatic soft tissue sarcoma, without evidence of residual disease (NCT01141491). The primary endpoint of PFS revealed no statistically significant difference between treatment groups; however, serologic responses were seen in the vaccine arm versus placebo (98 versus 21%) and minimal toxicities were described [11]. Additional studies using conjugated ganglioside vaccines will further define their role in sarcoma therapy.

Essentially all synovial sarcomas contain *t*(*X*; 18) representing the fusion of SYT (at 18q11) with either SSX1 or SSX2 [12]. SYT-SSX derived peptide vaccines were evaluated in twenty-one patients with advanced synovial sarcoma. Interferon-*α* was given as an adjuvant agent. While nine patients showed an increase in cytotoxic T-cells, only one patient had a decrease in tumor size [13].

Although attempts to use adjuvants such as GM-CSF to increase the immune system response have not yet translated to increased efficacy [14, 15], an ongoing phase I trial is exploring the role of the antigen bi-shRNAfurin and GMCSF autologous tumor cell (Vigil*™*) vaccine in patients with Ewing's sarcoma (NCT01061840).

## 3. Checkpoint Blockade

### 3.1. CTLA-4

CTLA-4 was the first immune checkpoint receptor to be clinically targeted. Normally after T-cell activation, CTLA-4 is upregulated on the plasma membrane, leading to downregulation of T-cell function through a variety of mechanisms [16, 17]. CTLA-4 blockade has significant potential because inhibitory signal suppression can result in the generation of antitumor T-cell response. Ipilimumab is a human monoclonal antibody that binds CTLA-4. It is FDA approved for the treatment of metastatic melanoma [18]. In a pilot phase II study, six patients with synovial sarcoma were treated with ipilimumab 3 mg/kg every 3 weeks and restaged following 3 doses. The primary endpoint of the study was RECIST 1.0 response rate. Secondary endpoints included determination of the clinical benefit rate and evaluation of NY-ESO-1 specific immunity. Four patients completed all 3 doses, while 2 patients each received 1 and 2 doses due to clinical or radiologic progression. No RECIST responses were observed, and time to progression ranged from 0.47 months to 2.1 months. No evidence of serologic or delayed type hypersensitivity to NY-ESO was noted. Termination of the study occurred due to slow accrual and lack of immune response [19].

Ipilimumab has demonstrated overall survival in metastatic melanoma with response rates of 10–20% [20, 21]. Data from these studies confirm that clinical responses can be delayed, with some patients showing disease regression or stabilization only after weeks or months after therapy is complete. Given what has been learned from melanoma, studies of CTLA-4 inhibition in patients with sarcoma should ideally include PFS or OS as primary endpoints with incorporation of immune-related response criteria. It is now well known that patients receiving immunotherapy, including ipilimumab, may have a response after an initial increase in tumor burden. Immune-related response criteria are a set of innovative response criteria designed to capture these unique response patterns [22]. Therefore, it may be premature to rule out the role of CTLA-4 in sarcoma based on the study design of this pilot trial. Interval imaging studies should be interpreted with caution, as early disease progression may not translate to a lack of efficacy.

In a KIT-mutant GIST mouse model, it was found that the immune system contributed extensively to the antitumor effects of imatinib, a tyrosine kinase inhibitor. Imatinib therapy activated CD8+ T-cells and induced regulatory T-cell apoptosis within the tumor by reducing immunosuppressive enzyme expression [23]. The critical role of T-cells in the antitumor effects of imatinib and GIST is being further explored through a phase Ib/II study of ipilimumab with dasatinib, another tyrosine kinase inhibitor, for patients with soft tissue sarcoma with an expansion of GIST (NCT01643278).

### 3.2. PD-1

The programmed cell death protein 1 (PD-1) receptor has proven to be an effective immunological target in solid tumor malignancies including non-small-cell lung cancer (NSCLC), melanoma, and renal cell carcinoma (RCC) with response rates exceeding 40% in previously untreated melanomas [24, 25]. Response to therapy can be seen as early as 12 weeks [25]. The impressive and durable response to anti-PD-1/PD-L1 immunotherapy has been coupled with enhanced safety and adverse event profile. Severe immune-related toxicities such as grade 4 pneumonitis, hypophysitis, diarrhea, and hepatitis occur at a rate of less than 10% in phase 3 studies and are typically managed with corticosteroids [26].

The role of PD-1/PD-L1 in soft tissue sarcomas is currently being evaluated. An ongoing phase II study (SARC028) is exploring the role of the anti-PD-1 antibody Pembrolizumab in patients with advanced sarcoma. The primary outcome measure is objective response rates with assessments conducted at 8 weeks, up to 5 years. Secondary outcome measures will include adverse events, overall survival, progression-free survival, and response rate by immune-related response criteria (NCT02301039). The study will also investigate CD8+ T-cells in tumor tissues before and after treatment. Recent data evaluated the role of tumor-infiltrating lymphocytes preexisting in the tumor prior to anti-PD-1 blockade and its predictive value for antitumor response [27]. The study indicated that tumor regression after therapeutic PD-1 blockade requires preexisting CD8+ T-cells, negatively regulated by PD-1/PD-L1 mediated adaptive immune resistance. SARC028 will also address this key hypothesis.

The role of PD-L1 expression as a biomarker in sarcoma remains debatable [28]. There remain challenges for standardizing the testing of PD-1/PD-L1, due to differences between the various anti-PD-L1 immunohistochemistry assays and mRNA technologies. Each test requires registered staining platforms and uses different definitions of a “positive” test for PD-L1 expression. There remain gaps in our knowledge of the technical and clinical aspects of these tests. Future studies are necessary not only to standardize testing for PD-L1, but also to assess its use as a predictive biomarker in patients with soft tissue sarcomas.

Dual checkpoint inhibition through CTLA-4 and PD-1/PD-L1 blockade has been evaluated in melanoma. Postow et al. concluded that previously untreated patients with metastatic melanoma had significantly greater objective response rates and progression-free survival when treated with combined CTLA-4 and PD-1 therapy compared to CTLA-4 inhibition alone [29]. Notably, drug-related adverse events of grade 3 or 4 were reported in 54% of patients receiving combination therapy, compared with 24% of patients receiving ipilimumab monotherapy.

Additional clinical studies exploring the role of other immune checkpoint inhibitors targeting TIM3, LAG-3, 4-1BB, and BTLA as monotherapy and in combination may prove to be beneficial in the treatment of soft tissue sarcomas.

## 4. Adoptive Immunotherapy

Adoptive immunotherapy offers a selective approach to eliminating cancer cells by relying on specific T-cell responses. This method involves expansion of a specific T-cell population that will recognize a precise antigen. Autologous T-cells are collected, expanded, and genetically manipulated to alter the T-cell receptor phenotype [5]. During the process of T-cell expansion, simultaneous expansion of immunosuppressive T-cells (T regulatory cells) can occur which may decrease the efficacy of this approach. Thus, a conditioning regimen using chemotherapy is often used prior to adoptive immunotherapy. In one of the initial immunotherapy studies in sarcoma, patients were conditioned with cyclophosphamide and fludarabine, followed by adoptive transfer of autologous T-cells transduced with TCR against NY-ESO-1. Objective clinical response was seen in four of six patients with synovial cell sarcoma [30]. One patient achieved a durable response lasting 18 months.

A phase I study examining autologous T-cells and cyclophosphamide in treating patients with metastatic or surgically inoperable myxoid/round cell liposarcoma and synovial sarcoma has been completed (NCT01477021). Two other studies explored combination therapy using adoptive T-cell transfer of modified T-cells with biologic therapy, interleukin-2. The study by Mackall et al. combined priming with tumor-peptide pulsed dendritic vaccination followed by T-cell harvest via apheresis for patients with newly metastatic or recurrent high-risk pediatric sarcomas. Patients underwent cytoreductive chemotherapy followed by reinfusion of T-cells and treatment with various doses of interleukin-2. Patients receiving immunotherapy experienced minimal toxicity [31]. In an ongoing phase II study at the NCI (NCT01967823), patients with metastatic cancers expressing the NY-ESO-1 antigen undergo a nonmyeloablative lymphodepleting preparative regimen followed by anti-NY-ESO-1 reactive T-cell receptor retroviral vector transduced autologous peripheral blood lymphocytes. Patients subsequently receive up to a maximum of 15 doses of high-dose interleukin-2 therapy. The study is currently recruiting patients.

Adoptive transfer strategies also include natural killer cells. This approach already has enthusing preclinical evidence in sarcomas. Twenty-one patients underwent harvesting of cytokine-induced natural killer cells, inhibiting autologous tumor xenograft growth in mice and destroying allogeneic and autologous sarcoma cells* in vitro* [5, 32]. An ongoing phase I/II study (NCT01898663) combines adenovirus-transfected autologous dendritic cells engineered to express MUC1 and survivin with cytokine-induced killer cells for treatment of intermediate and high-grade sarcomas. Preclinical data in rhabdomyosarcoma showed that survivin-responsive conditionally replicating adenovirus regulated with multiple factors (Surv.m-CRAs) could effectively kill all populations of rhabdomyosarcoma cells, including both stem cells and their progeny [33]. In another early-phase study, repression of survivin led to increased sensitization of rhabdomyosarcoma cells to T-cell attack [34]. A case report also described dramatic results with the use of adoptive immunotherapy in a patient with epithelioid sarcoma, treated with expanded lymphocytes and natural killer cells. The treatment was well tolerated, without any notable side effects [35].

Chimeric antigen receptor (CAR) modified T-cell therapy has shown promise in hematologic malignancies [36]. A recent phase I/II study evaluated the role of CAR modified T-cell therapy in nineteen patients with HER2 positive bone sarcomas [37]. Four of the 17 evaluable patients had stable disease for 12 weeks to 14 months. Three of these patients had their tumor removed, with one showing over 90% necrosis. Median overall survival of all patients was 10.3 months and no dose-limiting toxicities were reported [37]. The phase I VEGAS study is currently recruiting patients with refractory or metastatic GD2 positive sarcoma not responsive to standard treatment. The study uses CAR T-cells and incorporates an antibody that recognizes GD2 and is expressed in a T-cell that recognizes varicella zoster virus. The cells also contain two costimulatory molecules (CD28 and OX40 genes) (NCT01953900).

Initial evaluations of the safety and efficacy of CAR T-cells in sarcoma patients set the stage for additional studies that combine adoptive immunotherapy with other immunomodulatory approaches to enhance the expansion and persistence of the T-cells and NK cells.

## 5. Future Horizons

One method of optimizing the efficacy of current immunotherapy technologies is through upregulation or modification of tumor cell antigens, increasing their immunogenicity* in vivo*. The demethylating agent decitabine has been shown to epigenetically modify sarcoma cells, effectively increasing the expression of various antigens and peptides that increase the probability of a targeted T-cell response [38]. Pollack et al. also described the upregulation of cancer-testes antigen expression with the use of decitabine in chondrosarcoma cell lines [39].

In another study, pretreatment of a patient-derived primary osteosarcoma cell line with the HDAC inhibitor entinostat led to enhanced overall cytotoxicity* in vitro* and inhibited tumor xenograft growth [5, 40]. There remain unanswered questions regarding the role of epigenetic elements with known immunotherapy modalities in the management of sarcoma. Likewise, combination therapy using multimodality immunotherapy certainly merits further exploration.

An abscopal effect has been described anecdotally, in patients with metastatic melanoma where the addition of radiotherapy to ipilimumab led to tumor response in nonradiated lesions in addition to the radiation lesion [41, 42]. In one case, anti-MAGEA3 antibodies were found on serologic testing, revealing an association between the abscopal effect and a systemic antitumor immune response [42]. Radiotherapy has the potential to overcome numerous mechanisms of tumor immune evasion and also produce tumor specific cytotoxic T-cells [43, 44]. Radiotherapy has also been shown to increase the expression of PD-L1 on tumor cells [45]. Early-phase data have further established the rationale for combining radiotherapy and immunotherapy [46–48]. Various clinical trials combining ipilimumab and radiotherapy in patients with melanoma are currently open (NCT01449279, NCT01565837, and NCT01497808). The innovative approach of using combination immunotherapy and radiotherapy as a noninvasive strategy may prove applicable to soft tissue sarcomas in the near future.

Lastly, major advancements in tumor cell whole exome sequencing (WES) have allowed the identification of endogenous reactive T-cells to previously unknown tumor antigens [49]. Through this technique, unique target antigens have been identified and corresponding T-cell receptors have been genetically engineered for insertion into the patients' lymphocytes [50]. Future trials using these approaches will offer truly personalized treatment which is critical for heterogeneous sarcoma tumors.

## 6. Conclusions

**Figure 1:**
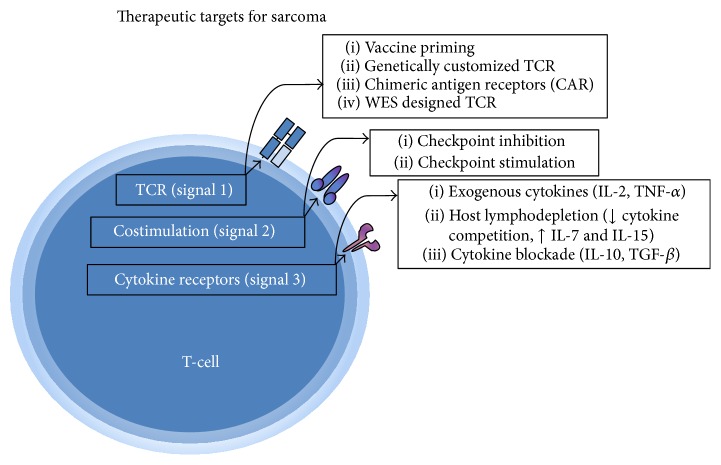
The stepwise signaling necessary for T-cell activation against tumor targets represents areas of active investigation for the immunotherapy of sarcoma (TCR: T-cell receptor; WES: whole exome sequencing).

The historic observations of sarcoma tumors inducing immune responses have matured into feasible and effective immunotherapies in use today. With a greater understanding of the process of T-cell activation [51], antitumor immunity against sarcoma is a reality with multiple avenues for future clinical application (Figure 1). Recent advances in combination immunotherapies and identification of novel tumor antigens by WES are areas of current investigation in sarcoma with a strong potential for clinical translation.
